# Progression of Oral Squamous Cell Carcinoma Accompanied with Reduced E-Cadherin Expression but Not Cadherin Switch

**DOI:** 10.1371/journal.pone.0047899

**Published:** 2012-10-23

**Authors:** Takashi Hashimoto, Yuichi Soeno, Genta Maeda, Yuji Taya, Takaaki Aoba, Masanori Nasu, Shuichi Kawashiri, Kazushi Imai

**Affiliations:** 1 Department of Biochemistry, The Nippon Dental University, Tokyo, Japan; 2 Department of Pathology, The Nippon Dental University, Tokyo, Japan; 3 Research Center for Odontology, The Nippon Dental University, Tokyo, Japan; 4 Department of Oral Surgery, Kanazawa University, Kanazawa, Japan; Okayama University, Japan

## Abstract

The cadherin switch from E-cadherin to N-cadherin is considered as a hallmark of the epithelial-mesenchymal transition and progression of carcinomas. Although it enhances aggressive behaviors of adenocarcinoma cells, the significance and role of cadherin switch in squamous cell carcinomas (SCCs) are largely controversial. In the present study, we immunohistochemically examined expression of E-cadherin and N-cadherin in oral SCCs (*n* = 63) and its implications for the disease progression. The E-cadherin-positive carcinoma cells were rapidly decreased at the invasive front. The percentage of carcinoma cells stained E-cadherin at the cell membrane was reduced in parallel with tumor dedifferentiation (*P*<0.01) and enhanced invasion (*P*<0.01). In contrast, N-cadherin-positive cells were very limited and did not correlate with the clinicopathological parameters. Mouse tongue tumors xenotransplantated oral SCC cell lines expressing both cadherins *in vitro* reproduced the reduction of E-cadherin-positive carcinoma cells at the invasive front and the negligible expression of N-cadherin. These results demonstrate that the reduction of E-cadherin-mediated carcinoma cell-cell adhesion at the invasive front, but not the cadherin switch, is an important determinant for oral SCC progression, and suggest that the environments surrounding carcinoma cells largely affect the cadherin expression.

## Introduction

The annual incidence of new cases of oral squamous cell carcinoma (SCC), worldwide, is estimated at 350,000 to 400,000 and is predicted to increase in the next few decades. Regardless of the therapeutic approaches and the location and stage of the diseases, >50% of patients experience a relapse [1]. Understanding molecular mechanisms regulating oral SCC progression is a prerequisite for improving the patient prognosis. At the first step of progression, SCC cells must sequester from their primary sites and invade into the basement membrane and underlying tissues. This step requires the dissociation of cell-cell adhesion. SCCs with weak cell-cell adhesion invade in a form of small subsets or individuals of cells, and predispose themselves to a more advanced state of progression. During the invasion, carcinoma cell phenotype and interaction with the microenvironments play a decisive role for the progression [2], [3]. From this standpoint, it is important to investigate the mechanism at the invasive front where the interactions occur.

Oral epithelial cells are connected to each other by tight cell-cell adhesion mediated by cadherins. Cadherins are calcium-dependent transmembrane proteins that are developmentally regulated and evolutionally conserved and form a superfamily. Among the cadherin superfamily, E-cadherin and N-cadherin are the most prominent members, and a large body of information about them has been collected [4]. E-cadherin is expressed in virtually all epithelial tissues, and N-cadherin predominantly in neural tissues but also fibroblasts, skeletal muscle, and endothelial cells [5]. Kan *et al*. [6] addressed the interchangeability by substituting E-cadherin gene (*CDH1*) to N-cadherin gene (*CDH2)* using the knock-in strategy in mice. Heterozygous mice co-expressing E-cadherin and N-cadherin show normal embryonic development and are viable. The homozygous knock-in embryonic stem cells form teratomas containing various epithelial-like structures. These results suggest that N-cadherin can support the formation of epithelia in the absence of E-cadherin.

During carcinoma progression, carcinoma cells at the invasive front frequently lose epithelial cell phenotypes and acquire mesenchymal cell-like phenotypes, referred to the epithelial-mesenchymal transition (EMT). The EMT enhances migratory, invasive and metastatic behaviors of carcinoma cells, and yields chemoresistance and stem cell-like features [7]. The reduction or loss of E-cadherin and the gain of N-cadherin expression, referred as the cadherin switch, are considered as a hallmark of EMT [7], [8]. The presence of cadherin switch and the clinical implications are well documented in adenocarcinomas of the gastrointestinal tract, breast and prostate [8]. However, expression of N-cadherin and the involvement in disease progression in SCCs are a controversial issue [9]–[12]. This study intends to examine the expression of E-cadherin and N-cadherin at the invasive front of oral SCCs and consider the expression in different cellular environments in plastic dishes and mouse tongue.

## Results

### Antibody Reactivity

**Figure 1:**
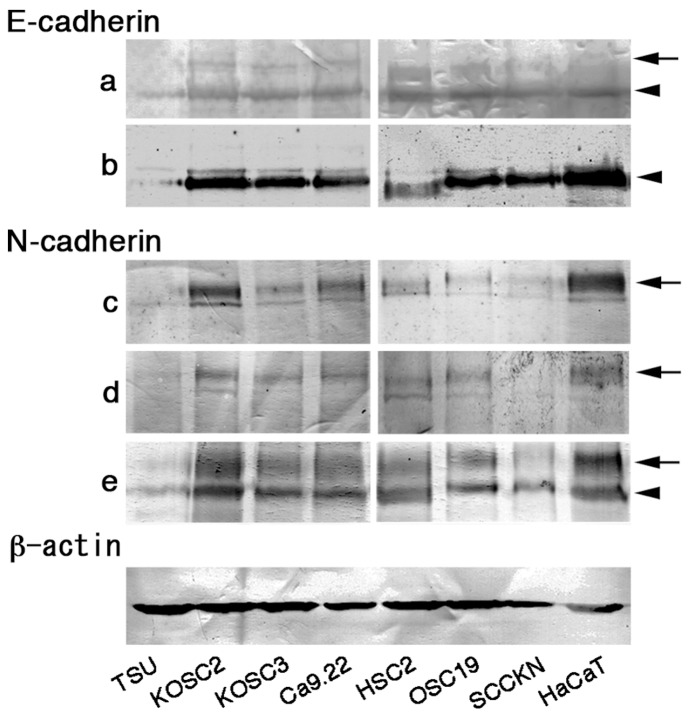
Reactivity of anti-cadherin antibodies. Reactivity of anti-E-cadherin and anti-N-cadherin antibodies was examined by the immunoblot. Antibodies against E-cadherin (a, Santa Cruz Biotechnology; b, R&D System) and N-cadherin (c, Invitrogen; d, Takara; e, LifeSpan Biosciences) were used. Arrows indicate a 125 kDa band and arrowheads a 120 kDa band. Antibody b and d were used for further experiments in this study.

E-cadherin (882 amino acids, 120 kDa under reduction) and N-cadherin (906 amino acids, 125 kDa under reduction) exhibit a high sequence homology of amino acids, and the molecular weights are post-transcriptionally modified by phosphorylation, glycosylation, ubiquitination and truncation [13]–[16]. Their molecular weights were monitored by the immunoblot analysis (Figure 1; Table S1). Several antibodies reacted 120 kDa and 125 kDa bands. Since all antibodies against E-cadherin and N-cadherin reacted 120 kDa and 125 kDa band, respectively, antibodies that reacted as a single band were selected to avoid difficulties in the data evaluation.

To verify the applicability for immunostaining on formalin-fixed and paraffin-embedded sections, cadherins were first stained on normal tissue slides (Figure S1). Anti-E-cadherin antibody reacted with the epithelial surface cells of the colon and stomach but not with cardiac muscle. Anti-N-cadherin antibody stained parietal cells of the stomach and intercalated discs of cardiac muscles but not the colon. These results were identical to a previous report [17]. Therefore, it was considered that these antibodies were applicable for the immunostaining on paraffin-embedded sections.

### Expression of Cadherins in Normal Oral Epithelium

**Figure 2:**
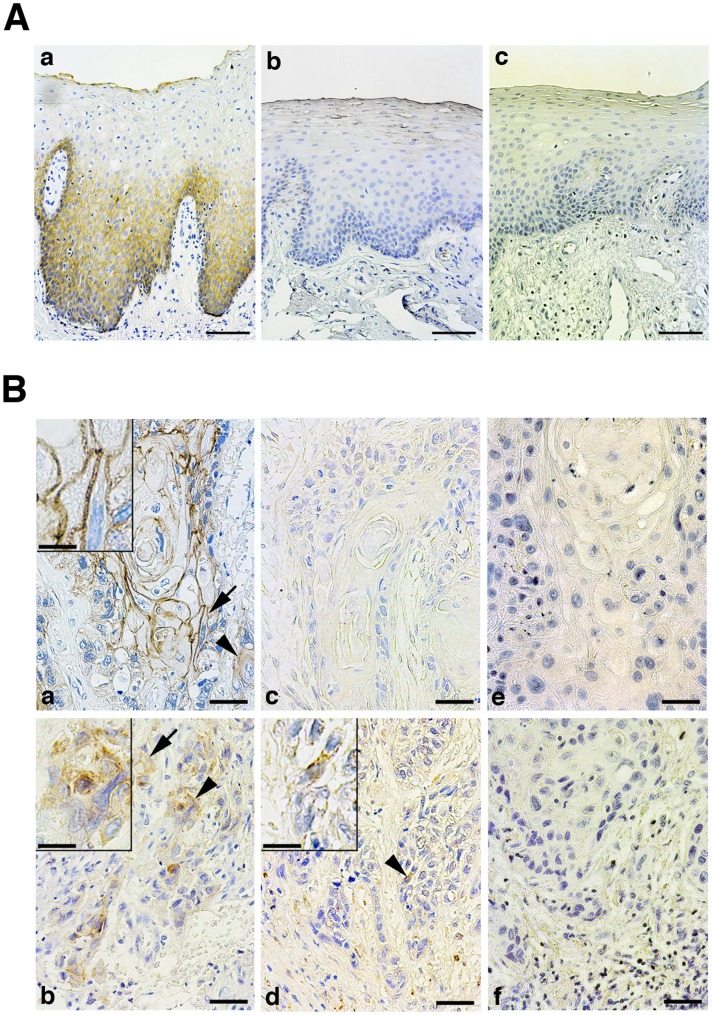
Localization of cadherins in normal oral epithelium and oral carcinoma tissues. A: E-cadherin was localized at basal and suprabasal cells of normal oral epithelium (a). N-cadherin-positive cells were not existed in the epithelium (b). Negative control using non-immune mouse IgG instead of primary antibody (c). *Bar* = 50 µm. B: Carcinomas at the center of tumor (a, c, e) and the invasive front (b, d, f) were stained by anti-E-cadherin (a, c) and anti-N-cadherin (b, d) antibodies. Cadherins were stained at cell membrane (arrows) or cytoplasm (arrowheads). Insets; high power view of cells pointed by an arrow (a) and arrowheads (b, d). e,f: negative control. *Bar* = 12.5 µm, and 4.3 µm (insets).

E-cadherin was immunolocalized at the cell membranes of basal and suprabasal epithelial cells (Figure 2A). Intensity of the staining in the suprabasal cells was much stronger than the basal cells, and gradually decreased or disappeared in cells that had undergone to keratinization. N-cadherin did not react with oral epithelial cells.

### Immunostaining of Oral SCCs and its Pathological Implications

The immunoreactivities were diverse from the location of cells at the center and the invasive front of tumors (Figure 2B). At the center of tumor masses, E-cadherin was strongly expressed and its localization mimicked the staining pattern in normal epithelium; the immunoreactivity was weak in cells located at the periphery of tumor cell nests as in the normal basal cells.

The E-cadherin-positive SCC cells at the invasive front rapidly decreased. Compared to the membranous staining at the center of tumor masses, it diffusely localized in the cytoplasm in a high frequency. Since cadherins are functional at adherence junctions but not when they are distributed to the cytoplasm, the percentage of positively stained SCC cells was independently calculated as the membranous stained group (36.7±34.4%, mean ± S.D.) and the cytoplasmic stained group (19.5±22.1%). Percentage of the membranous stained group was significantly decreased in parallel with the histological differentiation and the mode of invasion (Table 1). Although well-differentiated and low invasive (grades 1 and 2) SCCs showed the high percentage of membrane staining, poorly-differentiated and most invasive (grade 4D) SCCs dramatically reduced it.

**Table 1:** Percentage of E-cadherin immunoreactive cells at the invasive front and clinicopathological parameters.

Category	Subcategory	Cell membrane		Cytoplasm	
		Mean ± SD	*P* §	Mean ± SD	*P* §
T stage*	T1 (n = 16)	44.31±38.23	0.262	17.06±17.68	0.841
	T2 (n = 23)	35.20±35.20		22.96±24.18	
	T3 (n = 9)	18.56±29.38		18.22±26.15	
	T4 (n = 15)	45.13±36.91		17.60±21.87	
N stage*	N0 (n = 38)	44.92±37.21	0.214	20.63±21.12	0.885
	N1 (n = 16)	24.44±30.86		20.00±22.76	
	N2 (n = 8)	38.13±35.86		15.38±27.74	
	N3 (n = 1)	12.00		2.00	
Clinical stage*	Stage 1 (n = 14)	43.77±37.23	0.700	17.50±19.14	0.953
	Stage 2 (n = 19)	40.58±37.29		21.53±20.33	
	Stage 3 (n = 12)	26.00±34.26		18.92±24.24	
	Stage 4 (n = 18)	39.94±35.66		19.33±25.83	
Differentiation	Well (n = 26)	57.54±34.88	<0.001	29.62±25.37	0.002
	Moderate (n = 26)	31.73±32.12		14.65±18.11	
	Poor (n = 11)	8.55±18.11		7.09±9.45	
Invasion†	Grade 1 (n = 6)	60.00±30.04	<0.001	11.20±5.01	0.4186
	Grade 2 (n = 12)	36.00±35.46		26.00±27.88	
	Grade 3 (n = 26)	45.31±36.12		19.15±19.49	
	Grade 4C (n = 14)	30.56±36.17		19.00±27.24	
	Grade 4D (n = 5)	1.00±1.41		7.60±12.20	

* Patients were categorized by tumor size (T stage) and clinical stage according to the UICC WHO grading system and by the stage of lymph node metastasis (N stage).

† Patients were categorized by mode of invasion by Yamamoto *et al*. (1983).

§ Welch’s ANOVA.

N-cadherin immunoreactivities and the percentage of positively stained cells were limited. At the invasive front, the percentage of the membranous stained group and the cytoplasmic stained group were 3.17±4.40% and 0.92±1.41%, respectively. Neither of which were statistically associated with the clinicopathological parameters (Table S2). A positive relationship between both cadherins was observed at the invasive front in the membranous stained groups (*P*<0.05, data not shown). Although the significance for carcinoma progression was uncertain, fibroblast-like cells juxtaposed to SCC cells were frequently positively stained for N-cadherin as previously reported [18].

### Differential Expression of Cadherins in in vitro and in vivo Environments

**Figure 3:**
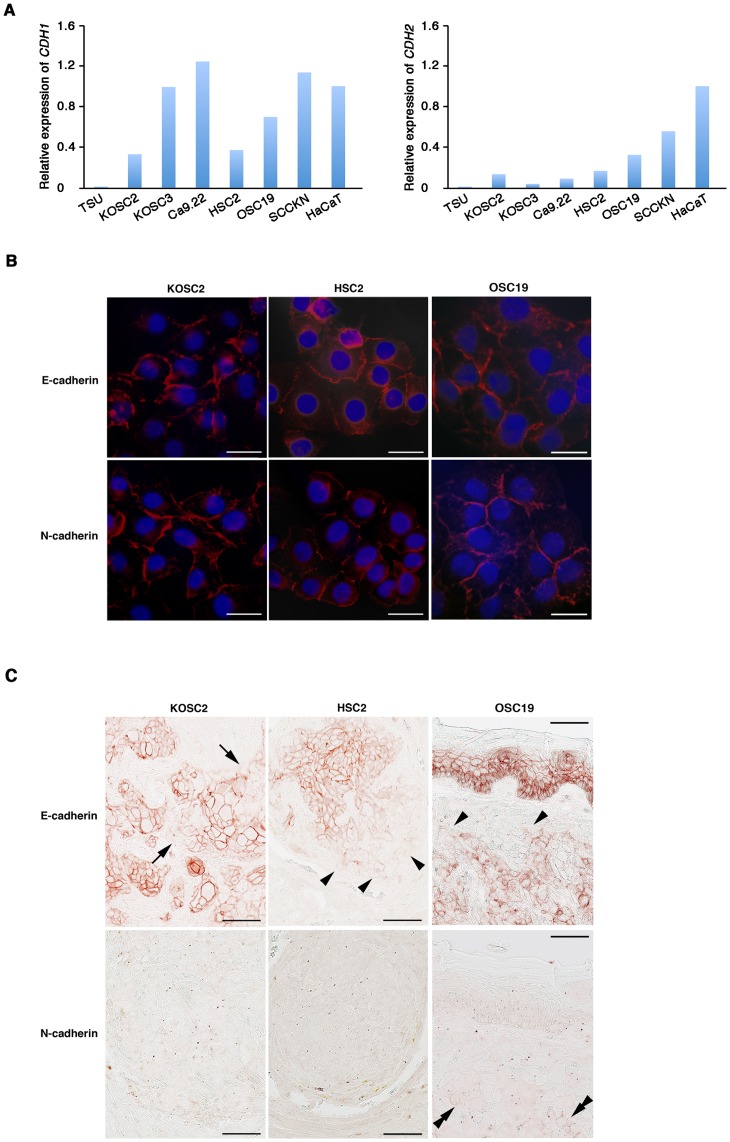
*In vitro* and *in vivo* cadherin expression in oral carcinoma cell lines. A: *CDH1* and *CDH2* expression were quantitatively examined by the real-time PCR. Relative expression was standardized by the expression level of *ß-actin* in each sample and calibrated with the expression in HaCaT cells. B: Oral carcinoma cells (KOSC2, HSC2 and OSC19) cultured on glass slides were stained with antibodies against E-cadherin or N-cadherin. *Bar* = 10 µm. C: Oral carcinoma cells were transplanted into the mouse tongue, and subjected to the cadherin immunostaining. Arrows indicate carcinoma cells at the peripheries of tumor cell nests; Arrowheads indicate carcinoma cells at the invasive front; Double arrowheads indicate N-cadherin-positive carcinoma cells. *Bar* = 12.5 µm.

Oral SCC cell lines cultured in plastic dishes apparently expressed E-cadherin and N-cadherin that were supported by the quantitative real-time PCR (Figure 3A). Since N-cadherin expression was very limited in oral SCC tissues, it was speculated that differential cellular environments play a decisive role for the expression. To verify the hypothesis, oral SCC cells expressing both cadherins were cultured on glass slides and subjected to the immunostaining and real-time PCR (Figure 3B and S2). Both cadherins strongly reacted in the cell membrane at the cell-cell contacts, and the mRNA was expressed at a comparable level to that on plastic dishes. When SCC cells were xenotransplantated into the nude mouse tongue, they developed tumors. Although E-cadherin was strongly detected in carcinoma cells, the reaction was reduced at the peripheries of tumor cell nests and at the invasive front cells (Figure 3C). As in oral SCC patients, little or no N-cadherin expression was detected. The negligible detection of N-cadherin is not attributed to the antibody sensitivity since the specific expression of E-cadherin in the tongue epithelium and N-cadherin in cardiac muscle was detected as in human normal tissue (Figure S3).

## Discussion

The cadherin switch from E-cadherin to N-cadherin strongly enhances aggressive phenotypes of adenocarcinoma cells and is expected to move the patients toward an unfavorable prognosis [8]. Advanced oral SCCs decreased E-cadherin expression but not upregulated N-cadherin. These findings were supported by a xenotransplantation mouse model showing that N-cadherin expression was negligible in tumor tissues regardless of its abundance in plastic and glass dishes. The reduction of E-cadherin expression, but not the cadherin switch in tissue environments, may regulate oral SCC progression.

E-cadherin plays an indispensable role for epithelial development and homeostasis and for tumor suppression. Targeting *CDH1* in the mouse skin promotes hyperproliferation of basal cells and the loss of terminal differentiation [19]. In a pancreatic cancer mouse model, adenomas convert to carcinomas with the loss of *CDH1* [20]. The loss of expression can result from inadequate protein regulation and gene expression [4]. Kudo *et al*. [21] reported the promoter hypermethylation of the gene in oral SCC cells at the invasive front but not at the non-invasive areas. In addition, the invasive front cells predominantly express the transcriptional repressors, SNAIL1 and ZEB2 [22], [23]. Protein dis-regulation also causes E-cadherin reduction. The p120^ctn^ binds to the cytoplasmic domain of E-cadherin, and protects from endocytosis and degradation of E-cadherin and from dissociation of cells [24]. Oral SCC cells at the invasive front decrease p120^ctn^ expression along with the E-cadherin reduction (Sasaya *et al*., manuscript in preparation). The present study showed that the expression of E-cadherin was maintained at the center of tumor masses and rapidly decreased towards the invasive front. The loss of expression was parallel with the dedifferentiation and the enhanced invasion. E-cadherin reduction at the invasive front may be regulated by a multi-faceted factor.

Recent studies emphasize a close association of inappropriate N-cadherin expression with carcinoma invasion and metastasis. However, most of this data was derived from adenocarcinomas [8]. Previous studies on oral SCCs reported that N-cadherin is expressed in a range of 37.0–92.4% of the cases, and expected that the cadherin switch may be involved in oral SCC progression [10], [25], [26]. However, the immunostaining data in this study showed that the percentage of N-cadherin-positive oral SCCs was limited and did not correlate with the clinicopathological parameters and E-cadherin reduction (data not shown). A recent study demonstrated that ∼90% of oropharyngeal SCCs were negative for N-cadherin and that cadherin switch had not happened [12]. Expression of N-cadherin is frequently observed in non-SCCs of the lung, but none of the lung SCCs express it [27]. Xenotransplantated mouse tumors in the present study confirmed negligible expression of N-cadherin. The presence and contribution of cadherin switch in SCCs may different from adenocarcinomas. Attention should be made to finally determine the role in the pathology of oral SCCs.

Aggressive subsets of oral SCC cells frequently lose the keratinocyte-differentiation markers and express mesenchymal cell-specific molecules at the invasive front [28]–[32]. This indicates the presence and involvement of EMT in the carcinoma progression. Although the cadherin switch is considered as a representable marker of the EMT, most studies on the role for aggressive behaviors of SCC cells were established by *in vitro* studies. Contradiction between *in vitro* and pathological studies makes understanding the role in SCC progression difficult. The cadherin switch in tissues, namely in a 3D environment, is a matter of debate [33]. In the present study, the xenotransplantation mouse model using carcinoma cell lines expressing both E-cadherin and N-cadherin *in vitro* reproduced the staining pattern in human oral SCC tissues: reduction of E-cadherin staining at the invasive front and the negligible N-cadherin-positive carcinoma cells. Although previous studies suggested the E-cadherin to P-cadherin switch in oral SCCs [25], [34], our preliminary immunoblot data suggests that P-cadherin and other cadherin expression are negligible (Figure S4). These facts strongly suggested that the cadherin switch *per se* is not involved in oral SCC progression. The reduction of E-cadherin expression at the invasive front highlights that tissue-factors strongly regulate the expression at the interface with carcinoma cells. Future studies on the cadherin regulation by cellular environments will expand understanding of the mechanism of oral SCC progression.

## Materials and Methods

### Patients

A total of 63 individual oral SCCs specimens were taken at Kanazawa University Hospital during incisional or excisional biopsy from 1988 to 2003. The median age of the study patients was 64.7 yrs (range, 39–93 yrs) at the time of diagnosis. The details of the pretreatment clinical and pathologic characteristics are summarized in Table 1. Histologic grading and staging were assessed according to the 1987 International Union Against Cancer (UICC) tumor-node-metastases classification. The mode of invasion classifies oral SCCs according to their histologic characters: grade 1, well-defined borderline; grade 2, cords, less marked borderline; grade 3, groups of cells, no distinct borderline; grade 4C, cord-like type with diffuse invasion; and grade 4D, widespread type with diffuse invasion [2]. Normal oral epithelium and other tissues were obtained from patients or autopsies who had no history of head and neck carcinomas. All tissues were obtained with the written consent and with approval by the institutional review boards of Kanazawa University and Nippon Dental University.

### Cell Lines

Immortalized human oral SCC cell lines (TSU, HSC2, KOSC2, KOSC3, SCCKN, OSC19, and Ca9.22) were obtained from the Cell Resource Center for Biomedical Research Institute of Development, Aging and Cancer (Tohoku University, Sendai, Japan) or RIKEN Cell Bank (Tsukuba, Japan) in 2010 and cryopreserved. The cell banks periodically characterize cell lines, cell morphology and karyotyping. Cells were maintained in 10% fetal bovine serum-containing DMEM or RPMI1640 medium (Sigma-Aldrich, St. Louis, MO) in a 5% CO_2_ incubator. Immortalized normal keratinocytes, HaCaT [35], were maintained in 10% fetal bovine serum-containing DMEM. All cells were cultured until 80–90% confluency was obtained.

### Orthotopic Nude Mouse Model of Oral SCCs

Female athymic BALB/c-nu/nu nude mice (CLEA Japan, Inc., Tokyo, Japan) were used at 7 weeks of age. They were housed and maintained in laminar flow cabinets under pathogen-free conditions. Oral SCC cells (KOSC2, HSC2 and OSC19; 2.0×10^5^ cells/25 µl of serum-free culture medium) were injected into the lateral tongue with a microsyringe with a 27-gauge needle as outlined in a previous study [36]. The mice were weighed twice a week and housed until 14 days after the injection. The mice were euthanized when they had lost more than 20% of their preinjection body weight or had become moribund. The tongue tumors were removed and fixed in formalin and embedded in paraffin. The mice were used in accordance with the Rules for the Care and Use of Laboratory Animals Guidelines of The Nippon Dental University under a protocol approved by the Institutional Review Board.

### Immunohistochemistry

Unstained formalin-fixed and paraffin-embedded sections of oral SCCs and normal tissues were treated with microwave (500 W, 12 min) in 0.01 M sodium citrate buffer, pH 6.0, and incubated (14 h at 4°C) with mouse antibodies against E-cadherin (25 µg/ml, clone 180215, R&D Systems, Minneapolis, MN) followed by biotinylated secondary antibodies (Vector Laboratories, Burlingame, CA). After treatment with avidin-biotin complexes (Vector Laboratories), the color was developed with 3,3′-diaminobenzidine tetrahydrochloride. For N-cadherin staining, sections were reacted with mouse antibodies to N-cadherin (10 ng/ml, clone N-cad 1–1–3, Takara, Ohtsu, Japan) for 14 h at 4°C and horse-radish peroxidase-labeled secondary antibody, and the color was developed using the CSA II staining kit (DAKO, Glostrup, Sweden). We determined that carcinoma cells with the strong circumferential membranous staining were membrane-positive and with strong cytoplasmic granular staining were cytoplasm-positive [37]. The percentage of carcinoma cells stained membrane and cytoplasm was independently determined according to previous studies [38]–[41]. All specimens were examined by two observers (T.H. and K.I.) blinded to the clinical and pathologic information. For each specimen, the numbers of carcinoma cells (over 3,000) and cadherin-positive carcinoma cells were counted by microscopic examination at x40 magnification. Subsequently, the percentage of the positive carcinoma cells to the total cells was calculated. To clarify the specificity of the staining, sections were reacted with non-immune mouse IgG (10 µg/ml) instead of primary antibodies.

In preparation for immunohistochemistry on mouse tissues, sections were incubated with mouse anti-E-cadherin (R&D Systems) or anti-N-cadherin antibodies (Takara) over night at 4°C, and the reaction was processed using a Mouse-on-Mouse staining kit (Vector Laboratories). After treatment with avidin-biotin complexes, the color was developed with AEC (Nichirei, Tokyo, Japan).

### Immunocytochemistry

KOSC2, HSC2 and OSC19 cells were cultured on glass slides (Lab-Tek Chamber II, Thermo Scientific, Yokohama, Japan) in 10% fetal bovine serum-containing medium and fixed in 3.7% paraformaldehyde for 15 min at 23°C. After treatment with 0.1% Triton X-100 in PBS for 7 min, the cells were reacted to primary antibodies: E-cadherin or N-cadherin for 16 h at 4°C. Alexa Fluor 546 anti-mouse IgG (Invitrogen, Camarillo, CA) was used for secondary antibodies.

### Real-time PCR

Total RNA was extracted from cell lines and reverse transcribed to cDNA by MultiScribe Reverse Transcriptase (Applied Biosystems, Foster City, CA) and was subjected to real-time PCR using the StepOne Real-time PCR system (Applied Biosystems). PCR conditions was 95°C for 20 s followed by 40 cycles of 95°C for 1 s and 60°C for 20 s. The *CDH1*- and *CDH2*-specific TaqMan probes (*CDH1*, Hs01023894; *CDH2*, Hs00983056; Applied Biosystems) were used. Expression levels were normalized against *ß-actin* (TaqMan Endogenous Control Human *ACTB*, Applied Biosystems). Levels of gene expression (2^−ΔΔCt^) were determined by the standard curve method [42] and calibrated by the levels of HaCaT cells.

### Statistical Analysis

Association of percentage of cadherin-immunoreactive cells and clinicopathological parameters were analyzed by Welch’s ANOVA or regression analysis using JMP 7.0.1 (SAS Institute Inc., Cary, NC).

## Supporting Information

Figure S1 Immunostaining of cadherins in human normal tissues. Normal tissues (a-d, stomach; e and f, colon; g and h, heart) were stained by an anti-E-cadherin antibody (a, b, e and g) and an anti-N-cadherin antibody (c, d, f and h). *Bar* = 125 µm (a and c), 50 µm (e and f), 25 µm (g and h), and 12.5 µm (b and d).
(TIF)

Figure S2 Expression of *CDH1* and *CDH2* in carcinoma cells cultured on plastic and glass dishes. Expression of *CDH1* and *CDH2* mRNA in carcinoma cells cultured on plastic dishes (blue bards) or glass dishes (green bards) were quantitatively measured by the real-time PCR using TaqMan probes.
(TIF)

Figure S3 Immunostaining of cadherin in mouse tissues. Normal tongue epithelium and cardiac muscles of mouse were stained for E-cadherin and N-cadherin. *Bar* = 12.5 µm
(TIF)

Figure S4 Immunoblotting for cadherins. Total cell lysates of oral SCC cells were loaded on SDS-PAGE gel and subjected to immunoblotting for P-cadherin, OB-cadherin and VE-cadherin.
(TIF)

Table S1 A list of anti-cadherin antibodies.
(DOC)

Table S2 Percentage of N-cadherin immunoreactive cells at the invasive front and clinicopathological parameters. ^*^Patients were categorized by tumor size (T stage) and clinical stage according to the UICC WHO grading system and by the stage of lymph node metastasis (N stage). ^†^Patients were categorized by mode of invasion by Yamamoto *et al*. (1983). ^§^Welch’s ANOVA
(DOC)

## References

[1] Choi S, Myers JN (2008) Molecular pathogenesis of oral squamous cell carcinoma: Implications for therapy. J Dent Res 87: 14–32. 18096889 10.1177/154405910808700104

[2] Yamamoto E, Kohama G, Sunakawa H, Iwai M, Hiratsuka H (1983) Mode of invasion, bleomycin sensitivity, and clinical course in squamous cell carcinoma of the oral cavity. Cancer 51: 124–129. 6189571 10.1002/1097-0142(19830615)51:12<2175::aid-cncr2820511205>3.0.co;2-m

[3] Hanahan D, Weinberg RA (2011) Hallmarks of cancer: the next generation. Cell 144: 646–674. 21376230 10.1016/j.cell.2011.02.013

[4] Imai K, Maeda G, Chiba T (2012) Cadherin expression and progression of head and neck squamous cell carcinomas. In: Li X, editor. Squamous Cell Carcinoma. Rijeka: InTech-Open Science. 121–136.

[5] Matsuyoshi N, Imamura S (1997) Multiple cadherins are expressed in human fibroblasts. Biochem Biophys Res Commun 235: 355–358. 9199196 10.1006/bbrc.1997.6707

[6] Kan NG, Stemmler MP, Junghans D, Kanzler B, de Vries WN, et al (2007) Gene replacement reveals a specific role for E-cadherin in the formation of a functional trophectoderm. Development 134: 31–41. 17138661 10.1242/dev.02722

[7] Thiery JP, Acloque H, Huang RYJ, Nieto MA (2009) Epithelial-mesenchymal transitions in development and disease. Cell 139: 871–90. 19945376 10.1016/j.cell.2009.11.007

[8] Wheelock MJ, Shintani Y, Maeda M, Fukumoto Y, Johnson KR (2008) Cadherin switching. J Cell Sci 121: 727–735. 18322269 10.1242/jcs.000455

[9] Gasparotto D, Polesel J, Marzotto A, Colladel R, Piccinin S, et al (2011) Overexpression of TWIST2 correlates with poor prognosis in head and neck squamous cell carcinomas. Oncotarget 2: 1165–1175. 22201613 10.18632/oncotarget.390 PMC3282075

[10] Nguyen PT, Kudo Y, Yoshida M, Kamata N, Ogawa I, et al (2011) N-cadherin expression is involved in malignant behavior of head and neck cancer in relation to epithelial-mesenchymal transition. Histol Histopathol 26: 147–156. 21154228 10.14670/HH-26.147

[11] Di Domenico M, Pierantoni GM, Feola A, Esposito F, Laino L, et al (2011) Prognostic significance of N-cadherin expression in oral squamous cell carcinoma. Anticancer Res 31: 4211–4218. 22199283

[12] Ukpo OC, Thorstad WL, Zang Q, Lewis JS Jr (2012) Lack of association of cadherin expression and histopathologic type, metastasis, or patient outcome in oropharyngeal squamous cell carcinoma: a tissue microarray study. Head Neck Pathol 6: 38–47. 22072429 10.1007/s12105-011-0306-7 PMC3311946

[13] Behrens J, Bakaet L, Friis R, Winterhager E, Van Roy F, et al (1993) Loss of epithelial differentiation and gain of invasiveness correlates with tyrosine phosphorylation of the E-cadherin/beta-catenin complex in cells transformed with a temperature-sensitive v-SRC gene. J Cell Biol 120: 757–766. 8425900 10.1083/jcb.120.3.757 PMC2119534

[14] Shore EM, Nelson WJ (1991) Biosynthesis of the cell adhesion molecule uvomorulin (E-cadherin) in Madin-Darby canine kidney epithelial cells. J Biol Chem 266: 19672–19680. 1918074

[15] Fujita Y, Krause G, Scheffner M, Zechner D, Leddy HE, et al (2002) Hakai, a c-Cbl-like protein, ubiqutinates and induces endocytosis of the E-cadherin complex. Nat Cell Biol 4: 222–231. 11836526 10.1038/ncb758

[16] Vallorosi CJ, Day KC, Zhao X, Rashid MG, Rubin MA, et al (2000) Truncation of the beta-catenin binding domain of E-cadherin precedes epithelial apoptosis during prostate and mammary involution. J Biol Chem 275: 3328–3334. 10652321 10.1074/jbc.275.5.3328

[17] Tsuchiya B, Sato Y, Kameya T, Okayasu I, Mukai K (2006) Differential expression of N-cadherin and E-cadheirn in normal human tissues. Arch Histol Cytol 69: 135–145. 16819153 10.1679/aohc.69.135

[18] Mink SR, Vashistha S, Zhang W, Hodge A, Agus DB, et al (2010) Cancer-associated fibroblasts derived from EGFR-TKI-resistant tumors reverse EGFR pathway inhibition by EGFR-TKIs. Mol Cancer Res 8: 809–820. 20530582 10.1158/1541-7786.MCR-09-0460 PMC2891820

[19] Tinkle CL, Lechler T, Pasolli HA, Fuchs E (2004) Conditional targeting of E-cadherin in skin: insight into hyperproliferative and degenerative responses. Proc Natl Acad Sci U S A 101: 552–557. 14704278 10.1073/pnas.0307437100 PMC327185

[20] Perl AK, Wilgenbus P, Dahl U, Semb H, Christofori GA (1998) A causal role for E-cadherin in the transition from adenoma to carcinoma. Nature 392: 190–193. 9515965 10.1038/32433

[21] Kudo Y, Kitajima S, Ogawa I, Hiraoka M, Sargolzaei S, et al (2004) Invasion and metastasis of oral cancer cells require methylation of E-cadherin and/or degradation of membranous ß-catenin. Clin Cancer Res 10: 5455–5463. 15328184 10.1158/1078-0432.CCR-04-0372

[22] Yu CC, Lo WL, Chen YW, Huang PI, Hsu HS, et al (2011) Bmi-1 regulates Snail expression and promotes metastasis ability in head and neck squamous cancer-derived ALDH1 positive cells. J Oncol 2011:609259. Available: http://www.ncbi.nlm.nih.gov/pmc/articles/PMC2948925/?tool=pubmed. Accessed 2012 Apr 23. 10.1155/2011/609259 PMC2948925 20936121

[23] Maeda G, Chiba T, Okazaki M, Satoh T, Taya Y, et al (2005) Expression of SIP1 in oral squamous cell carcinomas: Implications for E-cadherin expression and tumor progression. Int J Oncol 27: 1535–1541. 16273209

[24] Liu H, Komiya S, Shimizu M, Fukunaga Y, Nagafuchi A (2007) Involvement of p120 carboxy-terminal domain in cadherin trafficking. Cell Struct Funct 32: 127–137. 18159125 10.1247/csf.07023

[25] Pyo SW, Hashimoto M, Kim YS, Kim CH, Lee SH, et al (2007) Expression of E-cadherin, P-cadherin and N-cadherin in oral squamous cell carcinoma: correlation with the clinicopathologic features and patient outcome. J Craniomaxillofac Surg 35: 1–9. 17296306 10.1016/j.jcms.2006.11.004

[26] Li S, Jiao J, Lu Z, Zhang M (2009) An essential role for N-cadherin and beta-catenin for progression in tongue squamous cell carcinoma and their effect on invasion and metastasis of Tca8113 tongue cancer cells. Oncol Rep 21: 1223–1233. 19360298 10.3892/or_00000345

[27] Zynger DL, Dimov ND, Ho LC, Laskin WB, Yeldandi AV (2008) Differential expression of neural-cadherin in pulmonary epithelial tumours. Histopatholgy 52: 348–354. 10.1111/j.1365-2559.2007.02952.x 18269586

[28] Mizunuma H, Miyazawa J, Sanada K, Imai K (2003) The LIM-only protein, LMO4, and the LIM domain-binding protein, LDB1, expression in squamous cell carcinomas of the oral cavity. Brit J Cancer 88: 1543–1548. 12771919 10.1038/sj.bjc.6600952 PMC2377121

[29] Miyazawa J, Mitoro A, Kawashiri S, Chada KK, Imai K (2004) Expression of mesenchyme-specific gene HMGA2 in squamous cell carcinomas of the oral cavity. Cancer Res 64: 2024–2029. 15026339 10.1158/0008-5472.can-03-1855

[30] Uraguchi M, Morikawa M, Shirakawa M, Sanada K, Imai K (2004) Activation of WNT family expression and signaling in squamous cell carcinomas of the oral cavity. J Dent Res 83: 327–332. 15044508 10.1177/154405910408300411

[31] Maeda G, Chiba T, Kawashiri S, Satoh T, Imai K (2007) Epigenetic inactivation of IkappaB kinase-alpha in oral carcinomas and tumor progression. Clin Cancer Res 13: 5041–5047. 17785555 10.1158/1078-0432.CCR-07-0463

[32] Chiba T, Maeda G, Kawashiri S, Kato K, Imai K (2009) Epigenetic loss of mucosa-associated lymphoid tissue 1 expression in patients with oral carcinomas. Cancer Res 69: 7216–7223. 19738055 10.1158/0008-5472.CAN-09-1140

[33] Wendt MK, Smith JA, Schiemann WP (2010) Transforming growth facto-ß-induced epithelial-mesenchymal transition facilitates epidermal growth factor-dependent breast cancer progression. Oncogene 29: 6485–6498. 20802523 10.1038/onc.2010.377 PMC3076082

[34] Bauer K, Dowejko A, Bosserhoff AK, Reichert TE, Bauer RJ (2009) P-cadherin induces an epithelial-like phenotype in oral squamous cell carcinoma by GSK-3beta-mediated Snail phosphorylation. Carcinogenesis 30: 1781–1788. 19654099 10.1093/carcin/bgp175

[35] Boukamp P, Petrussevska RT, Breitkreutz D, Hornung J, Markham A, et al (1998) Normal keratinization in a spontaneously immortalized aneuploidy human keratinocyte cell line. J Cell Biol 106: 761–771. 10.1083/jcb.106.3.761 PMC2115116 2450098

[36] Kawashiri S, Kojima K, Kumagai S, Nakagawa K, Yamamoto E (2001) Effects of chemotherapy on invasion and metastasis of oral cavity cancer in mice. Head Neck 23: 764–7671. 11505487 10.1002/hed.1109

[37] Cohen D, Lane B, Jin T, Magi-Galluzzi C, Finke J, et al (2007) The prognostic significance of epidermal growth factor receptor expression in clear-cell renal cell carcinoma: a call for standardized methods in immunohistochemical evaluation. Clin Genitourin Cancer 5: 264–270. 17553206 10.3816/CGC.2007.n.002

[38] Langer C, Ratschek M, Rehak P, Schips L, Zigeuner R (2004) Are heterogenous results of EGFR immunoreactivity in renal cell carcinoma related to non-standardized criteria for staining evaluation? J Clin Pathol 57: 773–775. 15220376 10.1136/jcp.2003.015743 PMC1770368

[39] Anbalagan M, Moroz K, Ali A, Carrier L, Glodowski S, et al (2012) Subcellular localization of total and activated Src kinase in African American and Caucasian breast cancer. PLoS ONE 7: e33017. 22457730 10.1371/journal.pone.0033017 PMC3310861

[40] Zhang JY, Wang Y, Zhang D, Yang ZQ, Dong XJ, et al (2010) ∂-catenin promotes malignant phenotype of non-small cell lung cancer by non-competitive binding of E-cadherin with p120ctn in cytoplasm. J Pathol 222: 76–88. 20593408 10.1002/path.2742

[41] Srinivasan R, Gillett CE, Barnes DM, Gullick WJ (2000) Nuclear expression of the c-erbB-4/HER-4 growth factor receptor in invasive breast cancer. Cancer Res 60: 1483–1487. 10749108

[42] Schmittgen DD, Livak KJ (2008) Analyzing real-time PCR data by the comparative *C_T_* method. Nat Protoc 6: 1101–1108. 10.1038/nprot.2008.73 18546601

