# Supplementary figures and images for: Progression of Oral Squamous Cell Carcinoma Accompanied with Reduced E-Cadherin Expression but Not Cadherin Switch
Source: PLoS One. 2012 Oct 23;7(10):e47899. doi: 10.1371/journal.pone.0047899 (PMC3479144; doi:10.1371/journal.pone.0047899)

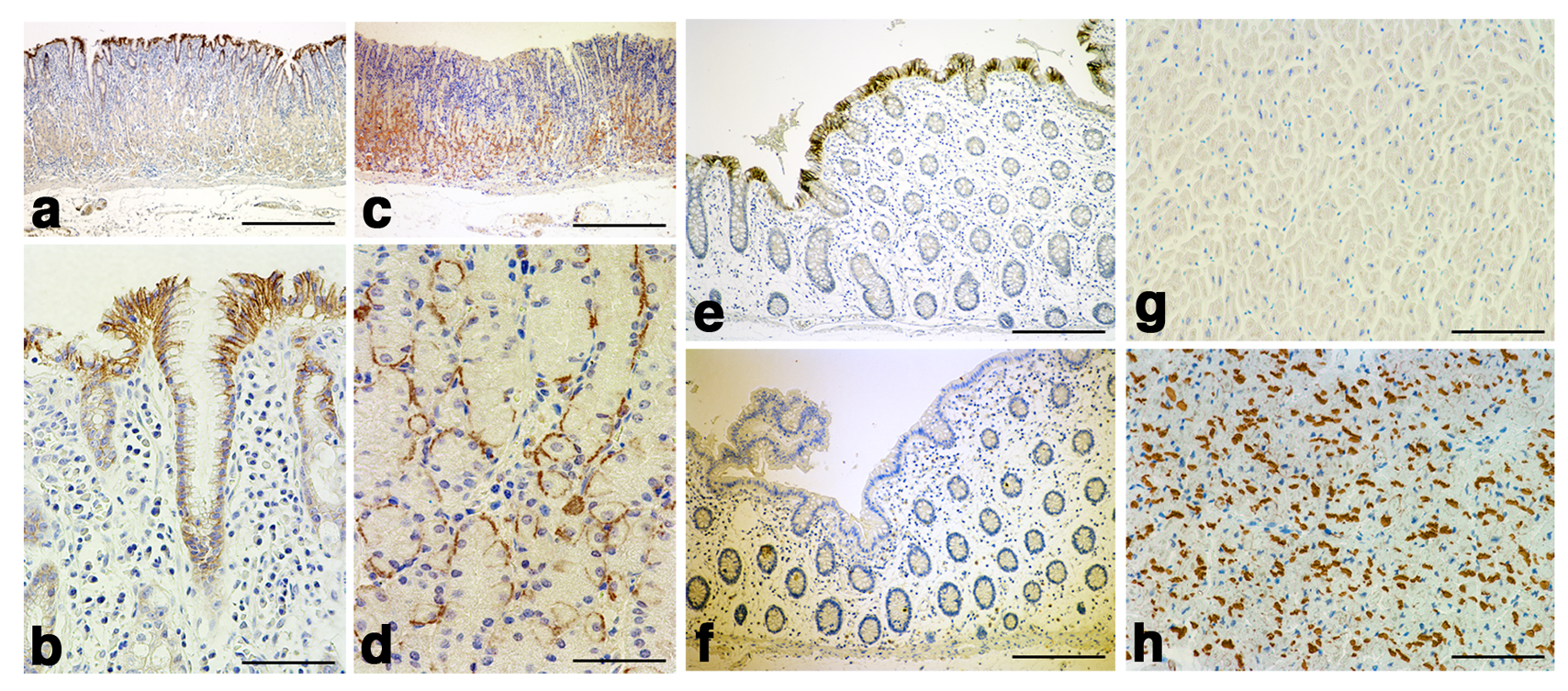

Supplement: Figure S1 — Immunostaining of cadherins in human normal tissues. Normal tissues (a-d, stomach; e and f, colon; g and h, heart) were stained by an anti-E-cadherin antibody (a, b, e and g) and an anti-N-cadherin antibody (c, d, f and h). Bar = 125 µm (a and c), 50 µm (e and f), 25 µm (g and h), and 12.5 µm (b and d). (TIF) [file pone.0047899.s001.tif]

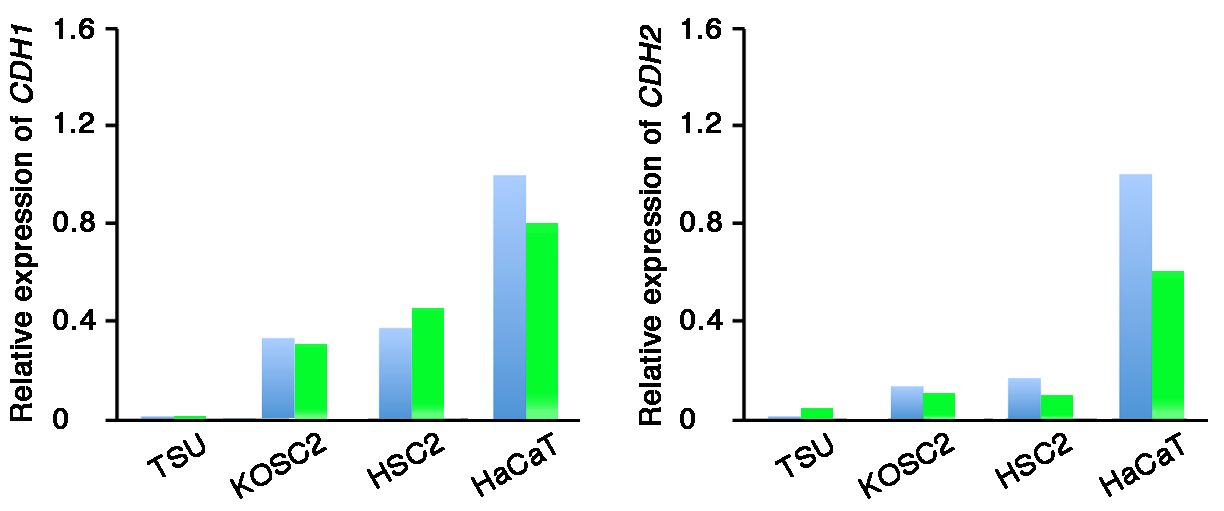

Supplement: Figure S2 — Expression of CDH1 and CDH2 in carcinoma cells cultured on plastic and glass dishes. Expression of CDH1 and CDH2 mRNA in carcinoma cells cultured on plastic dishes (blue bards) or glass dishes (green bards) were quantitatively measured by the real-time PCR using TaqMan probes. (TIF) [file pone.0047899.s002.tif]

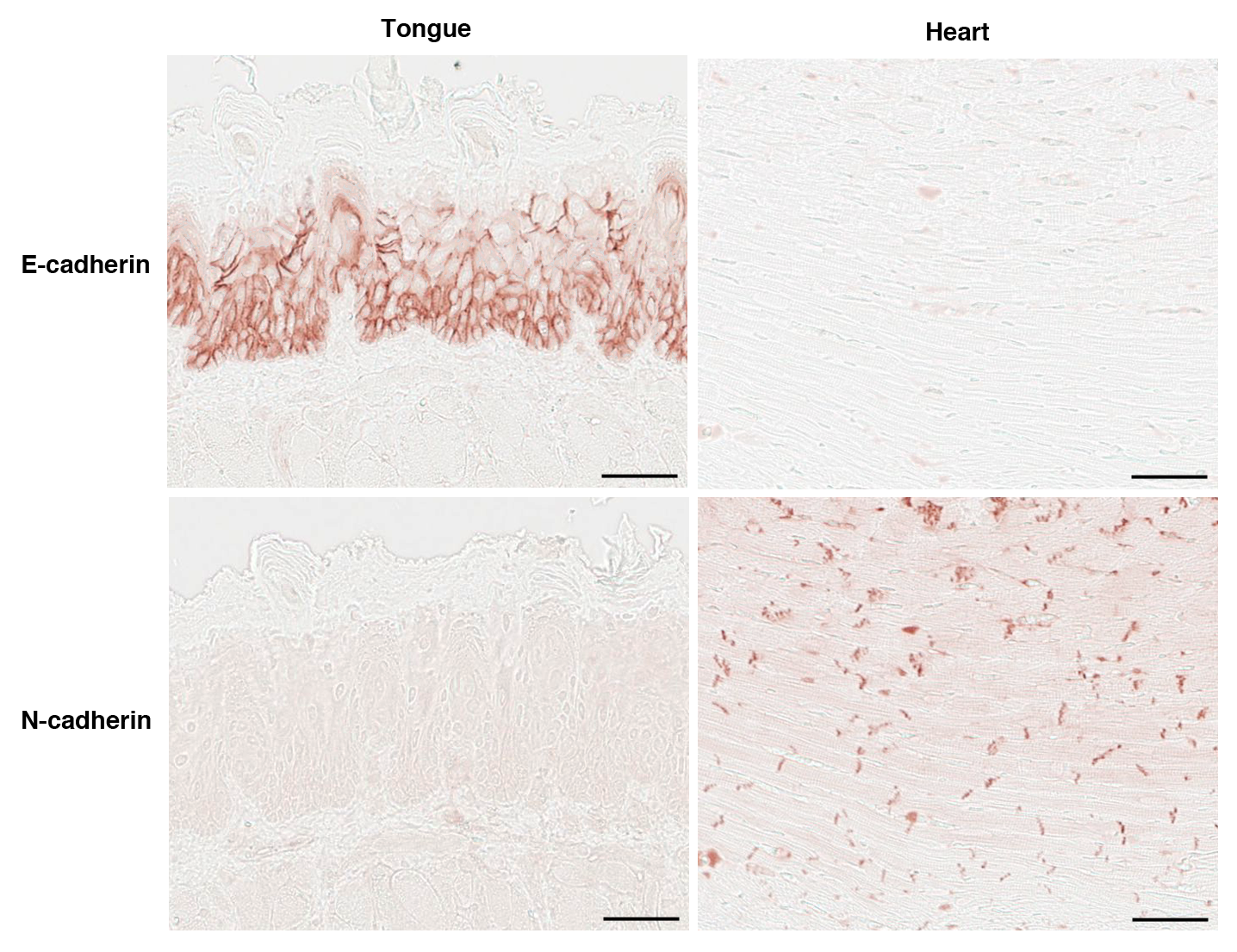

Supplement: Figure S3 — Immunostaining of cadherin in mouse tissues. Normal tongue epithelium and cardiac muscles of mouse were stained for E-cadherin and N-cadherin. Bar = 12.5 µm (TIF) [file pone.0047899.s003.tif]

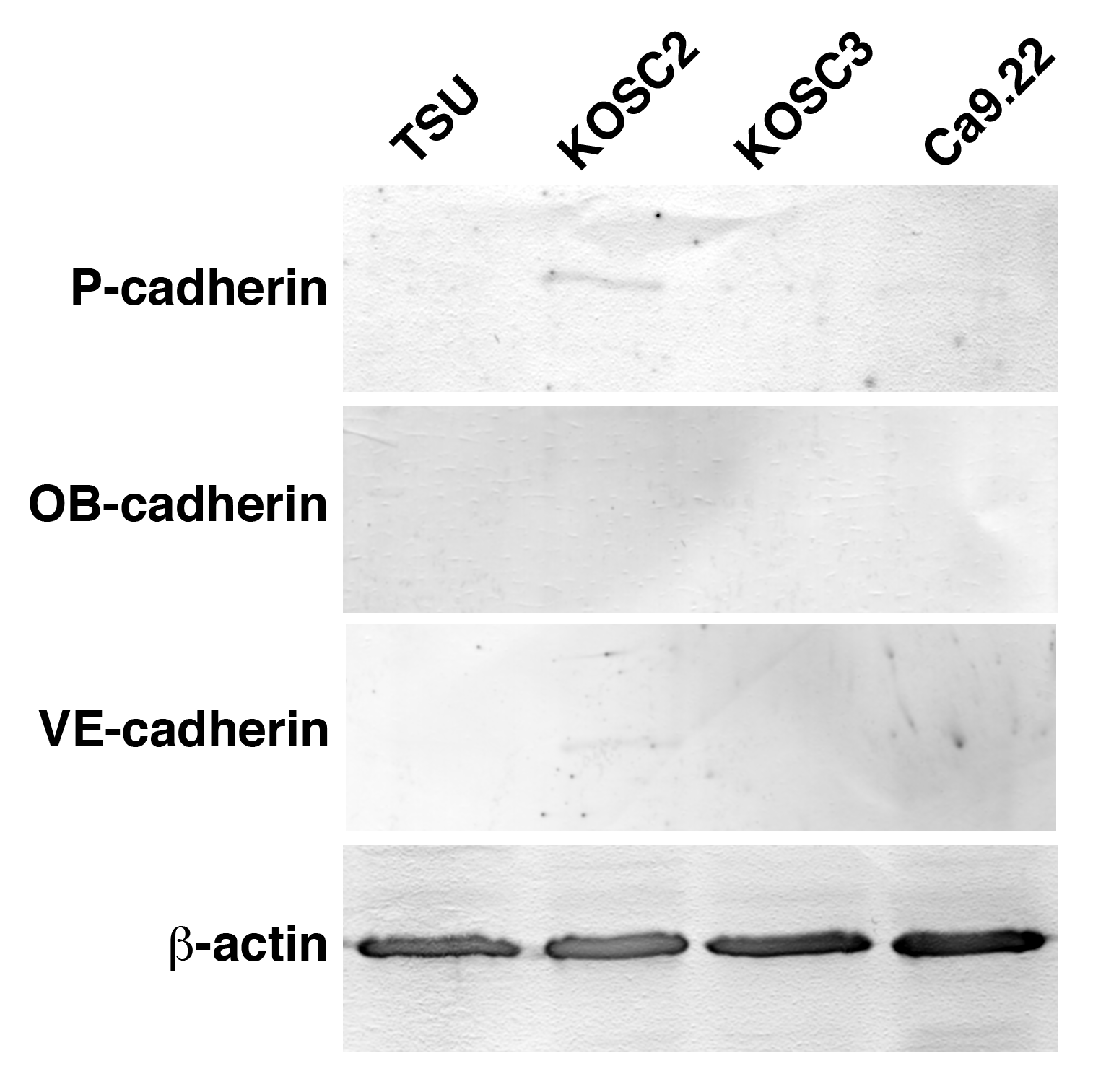

Supplement: Figure S4 — Immunoblotting for cadherins. Total cell lysates of oral SCC cells were loaded on SDS-PAGE gel and subjected to immunoblotting for P-cadherin, OB-cadherin and VE-cadherin. (TIF) [file pone.0047899.s004.tif]
