# Supplementary material for: Progression of Oral Squamous Cell Carcinoma Accompanied with Reduced E-Cadherin Expression but Not Cadherin Switch
Source: PLoS One. 2012 Oct 23;7(10):e47899. doi: 10.1371/journal.pone.0047899 (PMC3479144; doi:10.1371/journal.pone.0047899)
Supplement: Table S1 — A list of anti-cadherin antibodies. (DOC) [file pone.0047899.s005.doc]

**Table S1. A list of anti-cadherin antibodies.**

Company Clone Species Reference

Anti-E-cadherin antibodies

Santa Cruz Biotechnology sc-7870 rabbit Cancer Res 70: 7810-7819, 2010

R&D Systems 180215 mouse Int J Cancer 111: 484-493, 2004

Anti-N-cadherin antibodies

Invitrogen 3B9 mouse Mol Cell Biol 32: 1056-1067, 2012

Takara N-cad 1-1-3 mouse

LifeSpan Biosciences LS-B1080 rabbit
