# Supplementary material for: Progression of Oral Squamous Cell Carcinoma Accompanied with Reduced E-Cadherin Expression but Not Cadherin Switch
Source: PLoS One. 2012 Oct 23;7(10):e47899. doi: 10.1371/journal.pone.0047899 (PMC3479144; doi:10.1371/journal.pone.0047899)
Supplement: Table S2 — Percentage of N-cadherin immunoreactive cells at the invasive front and clinicopathological parameters. *Patients were categorized by tumor size (T stage) and clinical stage according to the UICC WHO grading system and by the stage of lymph node metastasis (N stage). †Patients were categorized by mode of invasion by Yamamoto et al. (1983). §Welch’s ANOVA (DOC) [file pone.0047899.s006.doc]

**Table S2. Percentage of N-cadherin immunoreactive cells at the invasive front and clinicopathological parameters**

Parameters Cell membrane Cytoplasm

Mean ± SD *P*^§^ Mean ± SD *P*^§^

T stage^*^ 0.19 0.11

T1 (n = 16) 3.44 ± 3.50 1.13 ± 1.59

T2 (n = 23) 2.30 ± 2.36 1.17 ± 1.64

T3 (n = 9) 4.67 ± 4.90 0.89 ± 1.17

T4 (n = 15) 5.60 ± 6.83 0.33 ± 0.72

N stage^*^ 0.15 0.07

N0 (n = 38) 3.32 ± 3.20 1.05 ± 1.00

N1 (n = 16) 2.50 ± 3.39 1.00 ± 1.60

N2 (n = 8) 8.50 ± 7.74 0.25 ± 0.71

N3 (n = 1) 0.00 0.00

Clinical stage^*^ 0.25 0.23

Stage 1 (n = 14) 3.86 ± 2.93 1.25 ± 1.46

Stage 2 (n = 19) 2.50 ± 3.39 1.37 ± 1.63

Stage 3 (n = 12) 3.75 ± 4.52 1.17 ± 1.62

Stage 4 (n = 18) 4.94 ± 6.51 0.56 ± 0.70

Histological differentiation 0.15 0.76

Well (n = 26) 3.58 ± 4.23 0.76 ± 1.03

Moderate (n = 26) 31.73 ± 32.12 1.03 ± 1.24

Poor (n = 11) 8.55 ± 18.11 1.00 ± 1.10

Mode of invasion^†^ 0.21 0.45

Grade 1 (n = 6) 4.40 ± 4.72 0.00 ± 1.42

Grade 2 (n = 12) 3.25 ± 3.08 1.17 ± 1.59

Grade 3 (n = 26) 4.77 ± 5.76 0.92 ± 1.41

Grade 4C (n = 14) 2.71 ± 2.58 1.21 ± 1.63

Grade 4D (n = 5) 1.40 ± 1.67 0.60 ± 0.89

^*^ Patients were categorized by tumor size (T stage) and clinical stage according to the UICC WHO grading system and by the stage of lymph node metastasis (N stage).

^†^ Patients were categorized by mode of invasion by Yamamoto *et al*. (1983).

^§^ Welch’s ANOVA
